# Genetic Association Study of *IL23R* and *IL12B* Polymorphisms with Psoriasis in a Romanian Population

**DOI:** 10.3390/life16040574

**Published:** 2026-04-01

**Authors:** Alessandra-Madalina Matei-Man, Ildiko-Orsolya Gaal, Andreea Catana, Stefan Vesa, Simona Senila, Elisabeta Candrea, Meda Orasan, Alexandra Puskas, Ana Calina Man, Teodora Mocan

**Affiliations:** 1 Dermatology Department, Faculty of Medicine, “Iuliu Hațieganu” University of Medicine and Pharmacy, 400126 Cluj-Napoca, Romania; madalina.man@outlook.com (A.-M.M.-M.); simonasenila@yahoo.com (S.S.); elisa_candrea@yahoo.com (E.C.); 2 Department of Medical Genetics, “Iuliu Hațieganu” University of Medicine and Pharmacy, Pasteur Street 6, 400349 Cluj-Napoca, Romania; orsigaal92@gmail.com (I.-O.G.); catanaandreea@gmail.com (A.C.); 3 Department 2 Functional Sciences, Discipline of Pharmacology, Toxicology and Clinical Pharmacology, Faculty of Medicine, “Iuliu Haţieganu” University of Medicine and Pharmacy, 400337 Cluj-Napoca, Romania; 4 Physiopathology Department, Faculty of Medicine, “Iuliu Hatieganu” University of Medicine and Pharmacy, 400126 Cluj-Napoca, Romania; meda2002m@yahoo.com; 5 Independent Researcher, 1021 Budapest, Hungary; dr.alexandradana@gmail.com; 6 Life Sciences, Biotechnology Department, Harvard Extension School, Cambridge, MA 02138, USA; anm7964@harvard.edu; 7 Physiology Department, Faculty of Medicine, “Iuliu Hatieganu” University of Medicine and Pharmacy, 400126 Cluj-Napoca, Romania; teodora_mocan@yahoo.com

**Keywords:** psoriasis, *IL23R*, rs11209026, *IL12B*, genetic polymorphism, IL-23/Th17 axis

## Abstract

**Background:** Psoriasis is a chronic immune-mediated inflammatory disease with a strong genetic basis, driven in part by the dysregulation of the IL-23/Th17 signaling axis. Variants in the *IL23R* and *IL12B* genes have been associated with psoriasis susceptibility; however, data from Eastern European populations remains scarce. **Objective:** We aimed to evaluate the link between *IL23R* rs11209026 and *IL12B* rs3213094 polymorphisms and psoriasis susceptibility in a multi-center, Romanian cohort. **Methods:** We performed a multi-center case–control study including adult patients with clinically and histopathologically confirmed moderate-to-severe psoriasis undergoing biological therapy and controls without autoimmune or chronic inflammatory diseases. Genotyping was performed using TaqMan SNP assays. Associations were analyzed under a dominant genetic model. **Results:** A significant association was observed between *IL23R* rs11209026 polymorphism and psoriasis susceptibility. Carriers of the A allele (AA+GA) showed reduced odds of psoriasis compared with the GG homozygotes, emphasizing the protective effect of this specific variant. No significant association was identified for *IL12B* rs3213094 polymorphism. **Conclusions:** Our findings support the protective association of *IL23R* rs11209026 A allele with psoriasis in a Romanian Eastern European population and emphasize the importance of the IL-23 pathway in disease pathogenesis. Alcohol consumption was independently associated with increased risk of psoriasis. Further studies are justified to explore potential pharmacogenetic implications.

## 1. Introduction

Psoriasis is a chronic, immune-mediated inflammatory disease of the skin with an estimated global prevalence of 2–4%, with a complex and multifactorial pathogenesis involving genetic, immunological, and environmental factors [1]. It is clinically characterized by erythematous plaques with overlying silvery scales, resulting from uncontrolled immune activation and keratinocyte proliferation. Traditionally considered a skin disorder, psoriasis is now recognized as a systemic inflammatory condition associated with multiple comorbidities, including psoriatic arthritis, metabolic syndrome, and cardiovascular disease. These associations reflect the systemic inflammation and persistent immune dysregulation in the disease’s spectrum. Advances in the understanding of psoriasis immunopathogenesis have shifted the perspective from viewing it as a primary epidermal disorder to a T-cell-mediated disease characterized by complex immune interactions between innate and adaptive immune pathways [2].

Central to disease pathogenesis is the IL-23/Th17 signaling axis. IL-23 stimulation promotes the differentiation, expansion, and maintenance of Th17 cells that secrete pro-inflammatory cytokines, including IL-17A, IL-17F, and IL-22, which drive keratinocyte proliferation. Mechanistic studies and clinical evidence demonstrate the therapeutic efficacy of monoclonal antibodies targeting IL-23 and IL-17 and support the relevance of this pathway. Furthermore, increased expression of IL-23R and IL-23A has been observed in psoriatic skin, correlating with disease severity and activity, thereby emphasizing the pivotal role of IL-23/Th17 signaling axis in psoriasis progression [2,3].

Genetic factors play an essential role in psoriasis susceptibility as well, with heritability accounting for approximately two-thirds of overall disease risk. GWAS studies and meta-analyses have identified over 100 susceptibility loci, several of which regulate immune pathways, including the IL-23/IL-17 signaling axis. These findings offer the basis for the development of targeted biologic therapies already in clinical use and highlight the role of cytokine signaling genes in disease risk. Among these loci, *IL23R* and *IL12B* are key genes within the IL-23 pathway, crucial for the differentiation of T-cells and inflammatory signaling. *IL23R* encodes the receptor for IL-23, while *IL12B* encodes the p40 subunit shared by IL-12 and IL-23 [4].

The *IL23R* rs11209026 polymorphism (Arg381Gln), as a functional coding variant, has been shown to attenuate IL-23–mediated signaling and downstream Th17 activation, making it particularly relevant for understanding disease mechanisms [5]. In contrast, many other variants within the IL-23 pathway are noncoding and demonstrate less consistent functional effects, highlighting the importance of studying variants with established biological relevance [6]. Genetic studies in Central and Eastern Europe, mainly including cohorts from Hungary and Poland, with genotyping performed from venous blood samples, have reported significant associations between *IL23R* polymorphisms and psoriasis susceptibility [7,8]. Similar findings have also been reported in broader European populations, including Spanish, Greek, and German cohorts [9]. In contrast, results for *IL12B* variants are less consistent, and variants such as rs3213094 have been less frequently studied as compared to other *IL12B* loci across populations [5]. However, overall data from this region remain limited, particularly regarding *IL23R* rs11209026 and selected *IL12B* polymorphisms. Differences in molecular approaches, such as tissue-based expression analysis and peripheral blood genotyping, may contribute to variability in findings, leading to the need for the use of standardized molecular techniques.

Studying Eastern European populations is particularly relevant, given that population-specific genetic architecture may contribute to differences in both susceptibility and treatment response [10]. In addition, genetic variation within cytokine signaling pathways may also influence the response to biologic therapy, supporting the rationale for pharmacogenetic investigations in psoriasis [11]. Patients treated with biologic agents represent an optimal population for pharmacogenetic investigation, as these therapies directly target key cytokine pathways central to psoriasis immunopathogenesis, particularly the IL-23/Th17 axis [10]. Despite their high efficacy, considerable interindividual variability in treatment response persists, suggesting that genetic determinants may partly explain differences in therapeutic outcomes [10,12].

Therefore, this multicentric case–control study aimed to assess the association between *IL23R* rs11209026 and *IL12B* rs3213094 genetic polymorphisms and psoriasis susceptibility in an Eastern European cohort, using TaqMan SNP assays for peripheral blood analysis. This study aims to contribute to the expansion of current knowledge and evidence of the genetic determinants of psoriasis in an understudied population.

## 2. Materials and Methods

### 2.1. Study Design and Population

This multicenter case–control study was conducted between August 2025 and February 2026 in Cluj-Napoca, Romania. Patients with psoriasis were recruited from the Department of Dermatology at the Emergency County Hospital Cluj as well as from a private dermatology practice in Cluj-Napoca. Consecutive patients were enrolled during the study period, and the final sample size consisted of all eligible individuals who met the predefined inclusion and exclusion criteria.

Control subjects were recruited from the Department of Dermatology at the Emergency County Hospital Cluj and consisted of patients hospitalized for non-autoimmune and non-chronic inflammatory dermatological or venereal diseases. Controls were matched by age (±2 years) and gender.

### 2.2. Inclusion and Exclusion Criteria

All patients included were 18 years or older and diagnosed with moderate-to-severe psoriasis vulgaris (requiring systemic therapy, including patients with BSA > 10%, involvement of special areas, or inadequate response to topical therapy, according to International Psoriasis Council consensus) [13]. Diagnosis was established clinically and confirmed by histopathological examination. At the time of inclusion, all patients were receiving biologic therapy (anti-IL-17A, anti-IL-23, or anti-TNF-α monoclonal antibodies).

Exclusion criteria for both groups included other autoimmune diseases, such as inflammatory bowel diseases and ankylosing spondylitis, given the shared genetic susceptibility and involvement of the IL-23/Th17 pathway across these immune- mediated disorders [14,15], and active malignancy.

### 2.3. Ethics Approval and Consent to Participate

The study was conducted in accordance with the Declaration of Helsinki. Written informed consent was obtained from all participants before inclusion. The study protocol was approved by the Ethics Committee of the “Iuliu Hațieganu” University of Medicine and Pharmacy, Cluj-Napoca (Approval No. AVZ 198, issued on 7 July 2025).

### 2.4. Clinical and Demographic Data Collection

Demographic and clinical data collected included age, sex, alcohol consumption (>1 drink/day), smoking status, hypertension, diabetes mellitus, cardiovascular disease (atherosclerosis or history of major cardiovascular events such as myocardial infarction, ischemic stroke, and documented coronary artery disease- including angina or coronary revascularization procedures), dyslipidemia, and body mass index (BMI). BMI was calculated as weight (kg) divided by height squared (m^2^), and patients were divided into normal weight, overweight, and obesity grades I–III [16]. Body mass index was calculated using objectively measured height and weight by a trained nurse following standard procedures. Body weight was assessed using a calibrated electronic medical scale (Seca GmbH & Co. KG, Hamburg, Germany), and height was measured with a fixed wall-mounted stadiometer. Dyslipidemia was categorized as hypercholesterolemia (total cholesterol levels > 200 mg/dL), hypertriglyceridemia (serum triglyceride level > 150 mg/dL), or mixed dyslipidemia according to contemporary ESC/EAS guidelines [17]. Participants not fulfilling these criteria were categorized as having no dyslipidemia. Laboratory parameters used to define dyslipidemia were assessed at the recruitment visit, prior to the initiation of biological therapy. In patients undergoing lipid-lowering treatment at the time of inclusion, lipid profile values before the initiation of therapy were retrieved from available medical records and considered as reference values for the analysis.

### 2.5. Sample Collection, DNA Extraction, and Genotyping

Peripheral blood was collected from the cubital vein into EDTA tubes under sterile conditions. Genomic DNA was isolated from whole blood using the PureLink Genomic DNA Mini Kit (Thermo Fisher Scientific, Van Allen Way, Carlsbad, CA 92008, USA), following the manufacturer’s protocol. DNA concentration and purity were assessed using a NanoDrop^TM^ spectrophotometer (Thermo Fisher Scientific, 5781 Van Allen Way, Carlsbad, CA 92008, USA), and only samples with an A260/A280 ratio between 1.8 and 2.0 were included for downstream analyses. Genotyping of the rs3213094 and rs11209026 single nucleotide polymorphisms (SNPs) was performed using predesigned TaqMan SNP Genotyping Assays (assay IDs: C_29927086_10 and C_1272298_10; Applied Biosystems, Waltham, MA, USA). The probe sequence for assay C29927086_10 was TAAAAGCTCAGGGTGTCGAGAACAA[C/T]GAACCAAGACTGTCATCCTGTCTCC, while for assay C__1272298_10 the probe sequence was ATTGGGATATTTAACAGATCATTCC[A/G]AACTGGGTAGGTTTTTGCAGAATTT. Reactions were carried out on a QuantStudio 3 Real-Time PCR System (Thermo Fisher Scientific, 5781 Van Allen Way, Carlsbad, CA 92008, USA) under the following cycling conditions: pre-read at 60 °C for 30 s; initial denaturation/enzyme activation at 95 °C for 5 min; followed by 40 cycles of denaturation at 95 °C for 15 s and annealing/extension at 60 °C for 90 s; and a post-read at 60 °C for 30 s. Approximately 5–10% of the samples were randomly selected and genotyped in duplicate to assess reproducibility, yielding a concordance rate of >99%. Moreover, negative controls (no-template controls) were included in each run to monitor potential contamination. Genotype calling was performed using TaqMan Genotyper Software v1.6 (Applied Biosystems, Carlsbad, CA, USA), and results were visually inspected using allelic discrimination plots to ensure accurate clustering and assignment.

### 2.6. SNP Selection and Rationale

The selection of *IL12B* and *IL23R* polymorphisms was based on prior evidence supporting their role as genetic susceptibility loci in psoriasis. Variants within both genes have been identified in genome-wide and candidate gene studies across different populations [18]. Among *IL12B* variants, rs3213094 has been previously associated with an increased risk of psoriasis, supporting its relevance as a candidate genetic marker [5]. Other *IL12B* variants, including rs3212227 and rs6887695, have been more extensively investigated in genetic studies of psoriasis [19]; however, rs3213094 was selected due to its reported association with psoriasis susceptibility [5]. Regarding *IL23R*, the rs11209026 polymorphism has been widely studied and shown to be associated with psoriasis susceptibility in meta-analyses and case–control studies [20] and has been confirmed as a susceptibility factor in genetic studies [21]. Based on this evidence, rs3213094 and rs11209026 were selected as representative polymorphisms within the *IL12B* and *IL23R* genes for analysis in the present study.

### 2.7. Statistical Analysis

Statistical analyses were performed using MedCalc^®^ Statistical Software version 23.4.9 (MedCalc Software Ltd., Ostend, Belgium; https://www.medcalc.org; 2026). Continuous variables were expressed as mean ± standard deviation (SD) and compared using Student’s *t*-test. Categorical variables were expressed as frequencies and percentages and compared using the chi-square test or Fisher’s exact test, whenever appropriate. Hardy–Weinberg equilibrium was assessed in the control group, and no significant deviation was found. Associations between genetic polymorphisms and psoriasis were evaluated under a dominant genetic model using logistic regression analysis, and odds ratios (ORs) with 95% confidence intervals (CIs) were calculated. The minor A allele of *IL23R* rs11209026 was rarely found in our study, that determined us to use a dominant genetic model (AA+GA vs. GG). Variables with *p* < 0.05 in univariate analysis were included in a multivariable logistic regression model. The limit of statistical significance was considered *p* < 0.05.

## 3. Results

### 3.1. Study Population Characteristics

A total of 154 participants were included in the study, comprising 103 patients with moderate-to-severe psoriasis vulgaris and 51 controls. Baseline characteristics are summarized in Table 1.

Alcohol consumption was significantly more prevalent in the psoriasis group. No statistically significant differences were observed between groups regarding smoking status, BMI distribution, dyslipidemia, diabetes mellitus, or cardiovascular disease.

### 3.2. Genotype Distribution and Association Analysis

Genotype distributions of *IL12B* rs3213094 and *IL23R* rs11209026 polymorphisms are presented in Table 2.

No significant association was identified between *IL12B* rs3213094 polymorphisms and psoriasis susceptibility under the genotypic model (*p* = 0.416). In contrast, *IL23R* rs11209026 polymorphism was significantly associated with psoriasis. In the dominant model for the A allele (AA+GA vs. GG), carriers of the A allele showed reduced odds of psoriasis compared with GG homozygotes (OR = 0.34, 95% CI: 0.12–0.97, *p* = 0.019).

### 3.3. Multivariable Logistic Regression

A multivariable logistic regression model was constructed to determine which variables that reached statistical significance in univariate analysis were independently associated with psoriasis. The overall performance of the model was assessed using the Hosmer–Lemeshow goodness-of-fit test. The model adequately fits the data (χ^2^ = 0.002, *p* = 0.963). The Nagelkerke pseudo-R^2^ was 0.136, which shows a modest explanatory power of the model. The dominant model of the *IL23R* rs11209026 SNP (AA+GA vs. GG) was significantly less frequent among patients. In contrast, alcohol consumption was independently associated with a higher likelihood of psoriasis (Table 3).

## 4. Discussion

Psoriasis is a chronic immune-mediated inflammatory disease characterized by dysregulated interactions between the innate and adaptive immune systems, with the IL-23/Th17 signaling axis playing a central role in disease pathogenesis [22]. Therefore, investigation of *IL23R* polymorphisms is directly relevant to understanding disease pathogenesis and genetic susceptibility.

In this multi-center case–control study, we explored the relation between *IL12B* rs3213094 and *IL23R* rs11209026 polymorphisms and psoriasis susceptibility in patients with moderate-to-severe psoriasis receiving biologic therapy. The results demonstrated a significant association between *IL23R* rs11209026 and psoriasis risk, with the A allele showing a protective effect. These findings are consistent with previous large-scale genetic studies in independent cohorts, reporting that the rs11209026 variant is associated with reduced psoriasis risk. In a U.S. cohort of 810 cases and 1256 controls, the minor allele was protective (OR = 0.56, 95% CI: 0.41–0.76) [23]. More recent genome-wide association studies further support the protective role of this variant across the psoriatic disease spectrum, including psoriatic arthritis [4]. Furthermore, a recent meta-analysis demonstrated a significant protective association of the rs11209026 A allele in psoriasis susceptibility (OR = 0.52, *p* = 0.002) [24]. However, most of the populations included in these studies were from Western Europe and North America. Results from Eastern European populations have been limited. Our findings in a Romanian population, therefore, provide geographic validations of this association.

There are also functional studies that provide the biological support for the protective effect of the A allele. The *IL23R* A/Gln381 allele has been shown to reduce IL-23-mediated signaling, resulting in reduced STAT3 phosphorylation and Th17 cell responses, including low production of IL-17A and IL-17F [25,26].

Genetic variations in the IL-23/IL-12 pathway may also influence disease phenotype and therapeutic response. In a study conducted by Eiris et al. in a Northern Spanish cohort, the rs11209026 GG variant was associated with increased disease severity (OR = 2.11) [19]. Although the focus of this study was not on PASI evaluation, the observed association between rs11209026 A and psoriasis susceptibility in our Romanian cohort is strengthened. Additionally, pharmacogenetic studies have suggested that genetic variation may influence response to TNF-α inhibitors. A case–control study reported that patients carrying the wild genotype achieved higher PASI90 rates under TNF-α blockade, while more recent meta-analytic evidence supports the role of genetic variants in modulating response to TNF inhibitors therapy [27,28]. However, pharmacogenetic findings remain heterogeneous, and no single genetic or biomarker has yet demonstrated sufficient predictive value for routine clinical implementation [29]. Real-world studies also suggest substantial variability in long-term biologic persistence, further supporting the potential role of pharmacogenetic biomarkers in predicting treatment outcomes [30].

Although large meta-analyses have consistently reported a protective role of the rs11209026 A allele, smaller studies in various populations have produced inconsistent results. A Turkish case–control study did not find a significant association between rs11209026 and psoriasis, despite investigating a geographically similar population. These discrepancies may reflect the differences in allele frequency, sample size, or underlying genetic background across populations [6].

The next gene of interest, *IL12B* rs3213094, showed no significant association with psoriasis susceptibility in our population. Previous studies investigating these polymorphisms produced inconsistent results. A pilot study conducted in a Mexican Mestizo population showed an increased risk for psoriasis among individuals carrying the C allele (*IL 12B* rs3213094), a result that was invalidated after correction for multiple testing [5]. Additionally, a large German case–control study reported significant associations of other *IL12B* polymorphisms with psoriasis vulgaris (OR around 1.5) [21]. Similarly, a case–control study in an Egyptian population did not demonstrate a significant association between *IL12B* variants and psoriasis susceptibility, although gene–gene interaction analyses suggested a combined effect with *IL23R* variants [31].

Alcohol consumption was independently associated with increased risk for psoriasis in our study group. Likewise, a meta-analysis, including 1.7 million subjects, reported a significant positive association between alcohol consumption and psoriasis (OR = 1.47, 95% CI 1.27–1.70), highlighting alcohol as a modifiable environmental risk factor in psoriasis pathogenesis [32]. In addition, several studies suggest that alcohol intake may contribute to psoriasis pathogenesis through multiple mechanisms, including stimulation of keratinocyte proliferation and disruption of the skin barrier. Ethanol metabolism generates reactive oxygen species, promoting an inflammatory environment and activating signaling pathways, leading to increased production of circulating proinflammatory cytokines—TNF-α [33]. Higher alcohol consumption has been reported among patients with psoriasis compared to the general population [34], and psychological stress and depression or anxiety may contribute to increased alcohol use as a coping mechanism [35]. This aspect is particularly relevant in Eastern European populations, where alcohol-related burden remains among the highest globally [36]. The high prevalence of alcohol use observed in our study may therefore be explained both by regional drinking patterns and by disease-related psychological burden.

Several limitations of this study should be acknowledged. First, the case–control design allows assessment of associations but does not allow causal inference and may be prone to selection bias. Second, the relatively modest sample size may have reduced statistical power. Third, all participants were recruited from tertiary dermatology centers and had moderate-to-severe psoriasis requiring biologic therapy, which may limit the generalizability of our findings to patients with milder disease. Additionally, our cohort consisted of individuals of similar ethnic backgrounds, which may limit the extrapolation of our findings to other populations. Another limitation is the absence of detailed phenotype stratification and treatment-response data, such as PASI score, which limited the evaluation of genotype–phenotype relationships and pharmacogenetic implications.

From a clinical perspective, although pharmacogenetic testing is not yet routinely indicated in psoriasis management, continued research into *IL23R* and related pathway variants may support future personalized therapeutic approaches. Integration of genetic biomarkers with clinical and immunological parameters may ultimately improve risk stratification and optimize biologic therapy selection in psoriasis.

## 5. Conclusions

In conclusion, we report an association between the GG genotype of rs11209026 in *IL-23R* and psoriasis susceptibility, and a protective effect of the A allele in the studied Romanian cohort. Additionally, alcohol consumption was independently associated with increased odds of psoriasis. Our results contribute to the genetic-specific landscape in a Romanian Eastern European population and further highlight the pivotal role of the IL-23/Th17 axis in psoriasis pathogenesis. Future research is justified to check if the studied polymorphisms could influence the response to biological therapy in the Romanian population.

## Figures and Tables

**Table 1:** Baseline characteristics of the study population.

Variable	Control (n = 51)	Psoriasis (n = 103)	*p*-Value
Age (mean ± SD)	50.1 ± 15.4	48.7 ± 14.5	0.594
Male sex, n (%)	34 (66.7%)	65 (63.1%)	0.799
Alcohol consumption, n (%)	14 (27.5%)	56 (54.4%)	**0.002**
Smoking, n (%)	20 (39.2%)	45 (43.7%)	0.597
Hypertension, n (%)	21 (41.2%)	36 (35%)	0.565
Diabetes mellitus, n (%)	8 (15.7%)	23 (22.3%)	0.333
Cardiovascular disease, n (%)	9 (17.6%)	13 (12.6%)	0.402
BMI categories			
Normal weight	13 (25.5%)	27 (26.2%)	0.984
Overweight	19 (37.3%)	40 (38.8%)	
Obesity grade I	11 (21.6%)	23 (22.3%)	
Obesity grade II	5 (9.8%)	9 (8.7%)	
Obesity grade III	3 (5.9%)	4 (3.9%)	
Dyslipidemia categories			
None	26 (51.0%)	42 (40.8%)	0.247
Hypercholesterolemia	11 (21.6%)	21 (20.4%)	
Mixed dyslipidemia	7 (13.7%)	29 (28.2%)	
Hypertriglyceridemia	7 (13.7%)	11 (10.7%)	

Bold values indicate statistical significance (*p* < 0.05).

**Table 2:** Association between *IL12B* and *IL23R* polymorphisms and psoriasis susceptibility.

Polymorphism	Genetic Model	Control (n = 51)	Psoriasis (n = 103)	*p*-Value
*IL12B* rs3213094	CC	32 (62.7%)	73 (70.9%)	0.416 *
	CT	14 (27.5%)	25 (24.3%)	
	TT	5 (9.8%)	5 (4.9%)	
*IL23R* rs11209026	AA+GA	9 (17.6%)	7 (6.8%)	**0.019**
	GG	42 (82.4%)	96 (93.2%)	

* *p*-value for *IL12B* rs3213094 was calculated using the chi-square test under the genotypic model. *p*-value for *IL23R* rs11209026 was obtained using univariate logistic regression under a dominant genetic model (AA+GA vs. GG). Bold values indicate statistical significance (*p* < 0.05).

**Table 3:** Multivariable logistic regression analysis of factors associated with psoriasis.

Variables	B	*p*	OR	95% CI for OR	
				Min	Max
*IL23R* rs11209026 AA+GA	−1.343	0.019	0.261	0.085	0.805
Alcohol consumption	1.269	0.001	3.558	1.665	7.603
Constant	0.310	0.270	1.364		

B, logistic regression coefficient; OR, odds ratio; CI, confidence interval; *p*, *p*-value.

## Data Availability

The data presented in this study are available on request from the corresponding author due to privacy.

## Abbreviations

The following abbreviations are used in this manuscript:

GWAS	Genome-Wide Association Study
BSA	Body Surface Area
SNP	Single Nucleotide Polymorphism
BMI	Body Mass Index
ESC/EAS	European Society of Cardiology/European Atherosclerosis Society
PASI	Psoriasis Area and Severity Index
